# Pozzolan Based 3D Printing Composites: From the Formulation Till the Final Application in the Precision Irrigation Field

**DOI:** 10.3390/ma14010043

**Published:** 2020-12-24

**Authors:** Nicola Schiavone, Vincent Verney, Haroutioun Askanian

**Affiliations:** Institut de Chimie de Clermont Ferrand (ICCF), UMR 6296 Université Clermont Auvergne, CNRS, SIGMA Clermont, F-63000 Clermont–Ferrand, France; nicola.schiavone@sigma-clermont.fr (N.S.); Vincent.Verney@uca.fr (V.V.)

**Keywords:** X-ray tomography, pozzolan, composites, material extrusion process, welding interfaces

## Abstract

A new eco-composite polymer for material extrusion fabrication based on fine fraction pozzolan waste was developed. In addition, the composite materials obtained were used to produce a self-watering pot with complex geometry and a permeable porous part to regulate the passage of water from the storage area to the roots of the plant. Moreover, the system was devised with a cover characterized by a UV-B barrier film. The results have shown the possibility of the 3D printing of complex geometric parts as microporous structures or thin films using a composite based on poly lactic acid (PLA) and pozzolan. The pozzolan has an effect of reinforcement for the composite and at the same time improves the cohesion between the layers of the part during printing.

## 1. Introduction

Additive manufacturing (AM) technologies are increasingly used for a wide range of new applications that require complex and multifunctional products. The commonly known AM technique is fused filament fabrication (FFF), which is part of the polymer material extrusion process family. FFF uses a solid polymer filament that is introduced and melted through a heated capillary and then deposited on a temperature-controlled bed. A 3D geometry is generated through the deposition of successive layers [1,2,3]. Different advantages have allowed the growth of this technology compared to traditional manufacturing processes. This technique offers the possibility of printing complex structures, the fabrication of objects already assembled, and the production of personalized products. Importantly, FFF enables the use of less materials compared to conventional manufacturing methods and has lower distribution emissions, which renders this technique an attractive environmentally sustainable manufacturing process [4,5]. 

In recent years, AM has found applications in the fields of agriculture, particularly in the advanced design and development of agricultural equipment [6,7,8]. The use of AM could be adopted for applications related to the context of precision agriculture. More specifically, the precision agriculture concept considers the use of technologies that are able to customize systems to gain higher efficiency, thus paying attention to ensuring a low environmental impact [9,10,11,12,13,14]. One interesting domain of precision agriculture is precision irrigation; in this domain, systems are used that control the required quantity of water to be supplied to the plants to function in the environmental condition and type of plant, which avoids water waste [9,10,11,12,13,14,15,16]. Another example of precision agriculture is the use of anti-UV polymer films widely adopted in the horticulture field. Researchers have been paying attention to the impacts of UV-B radiation (280–315 nm) on plants, since UV-B radiation has been recognized as a potential damaging factor for living organisms by modifying plant morphology, biochemistry, and development. This type of radiation induces growth retardation, macroscopic injuries, and oxidative damage for the plants [17,18,19,20,21]. Although there is strong interest in precision agriculture applications, there is a lack of scientific studies regarding the use of the material extrusion process in 3D printing in the horticulture field. Concurrently, the extensive use of polymeric materials for agricultural applications and the consequent generation of large quantities of waste, stresses the need for the sustainable development of agricultural systems. A promising method to reduce the amount of polymeric waste is the use of polymer composites charged with fillers [22,23]. Nevertheless, the processes of making solid filler are unsustainable and require significant amounts of non-renewable resources such as fossil fuels [24]. Considering the significant use of fillers in composite polymers, much attention has been paid to find more sustainable alternatives. A possible solution to decrease the environmental impact of the fillers is the direct use of unwanted by-products from industrial processes such as quarry waste. This valorization of waste offers additional benefits such as a reduction in CO_2_ emissions, increased profitability (increased income and reduced disposal costs), efficient use of resources, and improved sustainability for local communities [25]. 

In literature that has been developed for the field of new applications, new composite materials are available for the material extrusion process feedstock. For example, polymer composites have been formulated with carbon black, basaltic fibers, and Lecce stone. Moreover, the development of complex structures through AM technologies have mostly been studied in the field of construction using reinforced composite cement. However, studies on the effect of quarry waste fillers on the properties of polymeric composites for 3D printing is limited [26,27,28,29,30,31,32,33].

A quarry waste that has not yet been studied as a polymer filler is pozzolan. Pozzolan is a pyroclastic rock that is derived from mining, industrial, and agro-waste and is either in the form of clays or shales. [34,35]. It is an inorganic, poorly crystalline material that can be used directly from its natural origins or in artificial manufacturing. Natural pozzolan is rich in silicon dioxide and aluminum oxide and has a porous form and high thermal stability [36]. Pozzolan materials have been used for applications such as in construction building and roads, sanitation, and agriculture. In agriculture, it is used for drainage, soil amendments, substrate crops, and the restoration of soil [37,38,39,40]. In France, there are several quarries that produce pozzolan from volcanic origin with a large generation of pozzolan waste. Particularly in the Puy de Dôme and Haute-Loire regions, the pozzolan materials consist essentially of scoria and well stratified pumice stones. Its color varies from red to black, suggesting a relatively complex volcanological history. The chemical composition is divided between basaltic and trashy-andesitic pyroclastics [41,42,43]. 

In this work, we produced and analyzed a new eco-composite based on natural waste for 3D printing. We studied the effect of adding pozzolan waste to different PLA grades and its impact on the composite filaments for 3D printing. The pozzolan powder was obtained from the separation of the finer fraction following the extraction process from the quarry. Three PLA grades with different percentages of enantiomers have been used to produce six mixtures with varying pozzolan content. The thermal and rheological properties were analyzed by means of differential scanning calorimetry and rotational rheometry, respectively. The composite with the optimum properties was then selected to produce the filaments for 3D printing and generating pellets. Mechanical tensile and impact tests were conducted on the 3D printed and injected specimens. The morphological properties of the samples were analyzed with scanning electron microscopy and X-ray tomography. Finally, a smart irrigation pot with a porous structure controlling the water flow and a UV-B barrier film was designed and printed.

## 2. Materials and Methods 

### 2.1. Raw Materials

Pozzolan powder waste (PPW) was obtained from Pouzzolanes des domes S.A.S (Saint-Ours, France). The powder waste was a part of the storage on the quarry site and was obtained by a separation process after heating for 15 min at a temperature of 600 °C to remove the present moisture. It has a red color, and the chemical composition, according to the TAS (Total Alkali Silica) classification, is placed at the lower limit of trachybasalts. 

Commercial poly(lactic acid) resins (NatureWorks PLA Polymer 4042D, Natureplast PLE 005, NatureWorks PLA Polymer 4032D) (Minnetonka, MN, USA) were studied in this work. For simplicity, the PLA 4042D, PLE 005, and PLA 4032D are called PLA 1, PLA 2, and PLA 3, respectively, in this work. A summary of their properties as provided by the resin manufacturers is presented in Table 1. 

### 2.2. Laser Granulometry Measurement

Laser granulometry is a technique based on light diffraction using Fraunhofer’s theory. This test allows for particle size measurement with a radius between 0.05 and 900 μm and determines the size polydispersity in a sample [44]. In this study, the measures were carried out using a Malvern Mastersizer 3000 system. The particles are circulated and sonicated in water to carry out the analysis in liquid balk without particle agglomeration. The results were analyzed using the Mastersizer software to obtain the particle size and the cumulative volume distribution.

### 2.3. Preparation of Composite Pellets and the Filaments for 3D Printing

The preparation of composite pellets at different amounts of PPW (10%, 30%) was done with a twin screw extruder Thermo Fisher Scientific Pharma (Waltham, MA, USA) 11. Before the feeding of the materials in the extruder, the PLA and pozzolan were dried in an oven for 24 h at 60 °C. The process conditions were reported in Table 2. Each single PLA 1, PLA 2, or PLA 3 was mixed with 10% and 30% weight mass fraction of pozzolan powder. The sample codes were reported as follows: resin type-%PR, where %PR is the value of the pozzolan mass percentage. Composite pellets made with PLA 2 and PPW were chosen to produce the 3D printing filaments. The filaments obtained had a diameter of 2.85 mm.

### 2.4. Rheological Measurement

Shear dynamic measurements were carried out with an ARES rheometer (Rheometric Scientific) manufactured by TA Instruments (New Castle, DE, USA). The shear dynamic measurements with a frequency sweep method were conducted using parallel plate geometry with a diameter of 8 mm. The temperature used for the test was 190 °C and the gap was set to 1 mm. The frequency sweep was set to 0.1100 rad/s, and a strain of 5% was used. From the data, the loss viscosity (η′) and storage viscosity (η″) were calculated as a function of the frequencies. The zero shear viscosity η_0_ was determined from the experimental points since they can be obtained from the intersection of the arc of a circle with the abscissa, characteristic for a Cole–Cole distribution [45].

The study of the PPW effect on the volumetric contraction stress of the composites was analyzed with the force gap measurement as a function of the temperature and time with a force gap test in the sequence configuration. The gap was fixed at 1 mm and the temperature profile was characterized by a series of steps, each of 5 °C. The initial and final temperatures were 190 and 100 °C, respectively, and a duration of 2 min was set for each step [46].

### 2.5. Thermal Characterization

The crystallinity and thermal behavior of the studied samples were investigated by a differential scanning calorimetry (METTLER TOLEDO DSC 3+, Columbus, OH, USA) under a nitrogen atmosphere. For each sample, 8–10 mg of material was tested under a heat–cool–heat cycle method. The temperatures and heating/cooling rate were set from room temperature to 210 °C at 10 °C min^−1^. The percentage of crystallinity *X_c_* of the samples was calculated using the equation:

(1) $$X_{c}=\frac{\left|∆H_{m}\right|-\left|∆H_{c}\right|}{\left|∆H_{m}^{0}\right|\times {\left(w/w\right)}_{p}}\times 100$$

where Δ*H_m_*, Δ*H_c_*, and $∆H_{m}^{0}$ are the melting enthalpy, the crystallization enthalpy, and the melting enthalpy of pure fully crystalline polymers, respectively; and (*w*/*w*)*_p_* is the mass fraction of polymer in the matrix. The $∆H_{m}^{0}$ was taken as 93 J/g for PLA. To evaluate the evolution of the crystallization kinetics at the isothermal condition for each composite, an Avrami analysis was performed, where the thermal cycle was as follows: heating from 30 °C to 210 °C at 40 °C/min, 3 min at 210 °C to uniform temperature in the sample, then cooling down to a defined crystallization temperature (105 °C) at 40 °C/min. The crystallization half-time (t_1/2_) was calculated for each sample to compare the crystallization kinetics of the composites as a function of the PPW contents [47].

### 2.6. D Specimens and Smart Irrigation System Prototype Preparation

**Figure 1:**
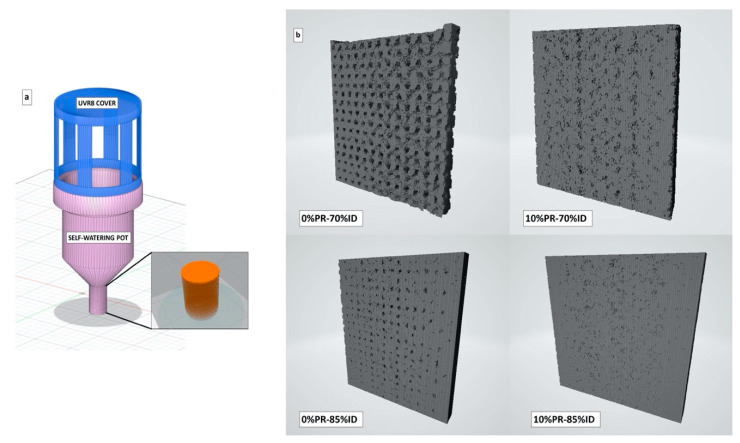
3D model of smart irrigation prototype (**a**) and a 3D model reconstruction obtained from X-ray tomography analysis of the permeable part 3D printed at different ID percentage (**b**).

An Ultimaker3 3D printer (Utrecht, Netherlands) was used to elaborate the specimens for mechanical tests and the prototypes of smart irrigation systems. The gcode files were elaborated through the CURA 4.1 software. The geometry of the specimens was selected according to ASTM D638 for the tensile test and ASTM D256 (West Conshohocken, PA, USA) for the Charpy impact test. All specimens were printed directly on the heating plate with a filling rate set to 100% and a linear infill pattern. All samples were printed in two different ways, the first way was simultaneous printing and the second was in sequence printing [48]. The smart irrigation prototypes were printed starting from the models developed with the 3D CAD software Autodesk Fusion 360. The system was conceived by assembling two parts. The first part was a self-watering pot designed in a way that the base of the container was characterized by a permeable porous structure (Figure 1). The porous structure was obtained by changing the infill density (ID) of the piece using a gyroid pattern geometry without walls. For the analysis of the porous structure effect on the liquid water flow, three print sets with different IDs were used: 70, 75, and 85%. The second part acted as a cover and was placed on the upper part of the container with the purpose of protecting the plant from UV-B radiation in the range of 280–315 nm. The geometry is shown in Figure 1a. The cover was printed separately and the printing direction was set starting from the top of the piece, therefore the model was rotated 180° with respect to the x-axis. The summary of the parameters is shown in Table 3. Furthermore, the specimens for mechanical studies were also produced with the injection molding process to compare the differences between the injected and 3D printed samples. The process conditions of the injection molding and 3D printing are reported in Table 4 and Table 5, respectively.

### 2.7. Mechanical Characterization

The tensile properties were evaluated according to ASTM D638 using a Lloyd EZ50 mechanical test machine (JLW Instruments, Chicago, IL, USA) at a crosshead speed of 30 mm/min. Tests were carried out at room temperature; at least five specimens were tested for each sample, and the results were averaged to obtain a mean value. The impact properties were also evaluated according to ASTM D256 using a Pendulum impact tester Zwick/Roell HIT (Ulm, Germany) with a pendulum of 50 J in Charpy configuration.

### 2.8. Morphology Characterization

The fractured cross-section from the tensile test printed samples was analyzed using a HIROX SH4000M scanning electron microscope (SEM) (Hirox Europe, Limonest, France). The coated samples were left to dry at room temperature before SEM could be performed. The SEM analyses were done at 20 kV and magnification equal to X41.

To analyze the morphological and porosity structure of the printed samples, a Skycan 1174 (Bruker, Billerica, MA, USA) X-ray radiography and tomography was used. Each sample was placed on a rotating plate while the X-ray beam passed through. The images were recorded by a CCD camera with a resolution of 1024 × 1024 pixels, which revealed different levels of X-ray absorption of the sample. The total exposure time for each sample was 450 s and the pixel size was 29.7 µm. Two images were taken per angular position and were averaged. After the reconstruction of the 3D structure part, the CT analysis software was used to measure the open/close porosity, specific surface of the voids, and to create a 3D model for the internal section of the permeable part of the pot. Furthermore, the isometric projections of the permeable part of the pot were obtained using DataViewer software 2.5.

### 2.9. Characterization of the Prototype System Functionalities

#### 2.9.1. Water Flow Measurement

The measurement of the water flow rate of the self-watering pot was carried out by measuring the volumetric flow rate of liquid as the hydrostatic pressure of the water in the container changes. The hydrostatic pressure was calculated starting from the measurement of the height of the liquid inside the container, in accordance with Stevin’s law. To keep the liquid level constant, the addition of water was made continuously to ensure minimum perturbation of the liquid bulk [49].

#### 2.9.2. UV–Vis Characterization

Ultraviolet-visible (UV–Vis) spectroscopy measurements were performed on the film part cuts by the 3D printed UVR8 cover where a Shimadzu UV-2600 spectrophotometer (Kyoto, Tokyo) was used. All measurements were done at room temperature.

## 3. Results and Discussion

### 3.1. Particle Size Distribution

The particle size distribution of the pozzolan powder was measured with a laser granulometry technique. The sizes at ten (D_10_), fifty (D_50_), and ninthly (D_90_) percent of cumulative volume were calculated. The obtained values were 16, 56, and 126 µm, respectively. The specific surface area was 2939 cm^2^/g. The distribution did not present polydispersity and the width of the narrow distribution (span) was 1.96, calculated as the ratio between the difference of the diameter at 90% and 10% of the cumulative volume and the diameter at 50% of the cumulative volume [50].

### 3.2. Thermal and Rheological Properties of the Composites

#### 3.2.1. Differential Scanning Calorimetry (DSC) Analysis

The effect of the PPW on the thermal behavior of the PLA composites was studied with a differential scanning calorimetry. The glass transition temperature (T_g_), melting temperature (T_m_), crystallization temperature (T_c_), crystallization enthalpy (*ΔH_c_*), and crystallinity degree (X_c_) of the composites are reported in Table 5. The addition of the pozzolan did not have a significant effect on the glass transition and melting temperature of the studied composites. The poly-lactide acid used in this work had a different percentage of D-enantiomers (Table 1) and this difference was reflected in their crystallization behavior. The PLA1 can be considered amorphous with only 1% of crystallinity degree, while the PLA2 and PLA3 are semicrystalline with a crystallinity degree around 14% and 11%, respectively [51]. Concerning the effect of the pozzolan on the degree of crystallinity, in the case of the composite PLA3/PPW, the crystallinity degree was significantly increased up to 38% by adding 10% of pozzolan fillers and 25% for 30% of pozzolan rate. A similar phenomenon has been reported in other studies, underlining the nature of the fillers as nucleation agents for PLA3; in contrast, the PLA1/PPW retained the amorphous nature of the polymer matrix without being affected by the filler [52,53,54]. Instead, the PLA2/PPW showed a slight dependence on the filler content, and a decrease in crystallinity degree x_c_ was observed from 14% for the pure PLA2 to 11% at 30% of pozzolan powder content.

The values of crystallization half-time were calculated for the composites by the Avrami method at the crystallization temperature of 105 °C. The results confirm what was observed for the degree of crystallinity, indeed, the PLA1/PPW and PLA2/PPW did not show a significant variation of the half-time of crystallization with the PPW content. Particularly, PLA1/PPW still had an amorphous comportment with a t_1/2_ around 2640 s. For the PLA2/PPW composites, the values of t_1/2_ were similar for both contents of PPW (10% and 30%) and included in the range of 240–245 s. Instead, PLA3/PPW showed a reduction in the crystallization rate with a value of t_1/2_ equal to 395, 173, and 128 s for the 0%, 10%, and 30% of PPW contents, respectively.

#### 3.2.2. Rheology

**Figure 2:**
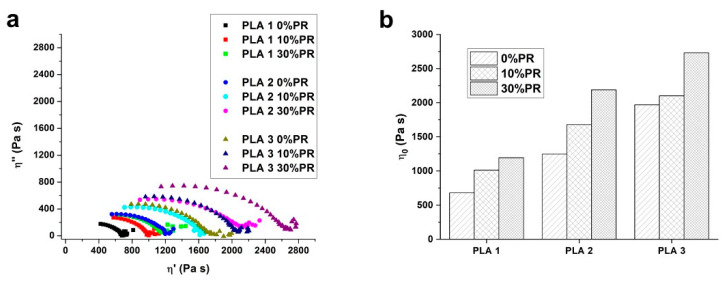
Cole–Cole plot (**a**) and Newtonian viscosity (**b**) for all the composites studied.

**Figure 3:**
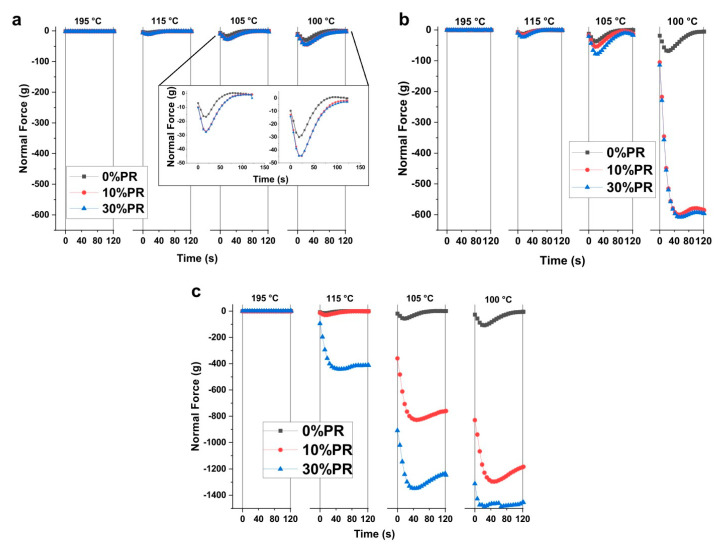
Normal force curves obtained with the constant gap test as a function of temperature and time for: PLA1/PPW (**a**), PLA2/PPW (**b**), and PLA3/PPW (**c**) composites.

The effect of PPW content on the viscoelastic properties of the composites was investigated by melt viscoelasticity experiments in oscillatory shear mode using a rotational controlled stress rheometer equipped with parallel plate geometry. The Cole–Cole plots of all samples are reported in Figure 2a. As the results show, the Cole–Cole distribution highlights a significant increase of the zero shear viscosity by increasing the PPW content in the composites (Figure 2b). The increasing effect of the viscosity after the incorporation of the filler in a polymer matrix is normally due to the hindered mobility of chain segments changing the normal polymer flow [55,56,57]. In other words, the presence of the pozzolan improves the viscoelastic properties and reflects good interfacial adhesion between the pozzolan fillers and PLA matrix. In parallel, the effect of the fillers on the volumetric contraction during the cooling of the composites was analyzed with the measurement of the normal force variation, monitored with the gap test at a constant gap. The rheograms related to the normal force measurement are reported in Figure 3. The plotted curves are relative at four temperatures chosen to focus on the most important areas of the curves. The results showed that the presence of the pozzolan increased the normal force evolution for all the studied samples; this effect varies according to the PLA grade used to elaborate the composites. In particular, the PLA1/PPW composite exhibited a slight increase in normal force, while the PLA2/PPW and PLA3/PPW composites showed an important increase, especially for PLA3/PPW. Moreover, the normal force increased by decreasing the temperature with a faster rate for the PLA3/PPW composites. This behavior is due to the formation of the crystalline phase during the cooling. In fact, the highest normal force was read for the PLA3/PPW composite, which, as observed by thermal analysis, had the highest crystallization degree and rate. From each curve, a maximum force value of retraction can be depicted. This characteristic of the materials can indicate that after each temperature change, there is a reorganization time of the polymer chains [46].

### 3.3. Characterization of the Printed and Injected Samples

**Figure 4:**
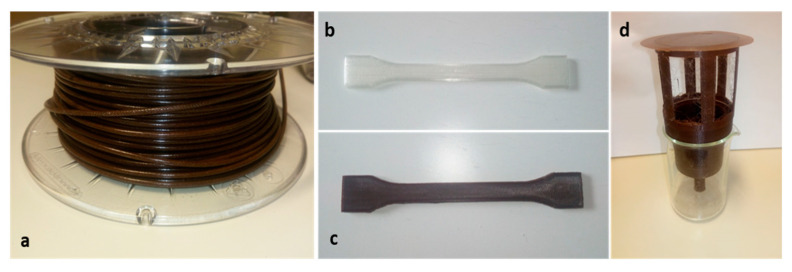
3D printing filament composite produced with PLA + PPW (**a**), 3D printing tensile specimens (**b**,**c**), smart irrigation system (**d**).

PLA2 was chosen as the polymer matrix for the 3D printed samples. The choice of this PLA was related to the optimum rheological and thermal properties. As noted, the PLA 2 based composite had an intermediate thermal and rheological behavior between the PLA1 and PLA3 composites. Figure 4 shows the filament obtained from PLA2/PPW for 3D printing (Figure 4a), the 3D printed samples for tensile tests (Figure 4b,c), and a smart irrigation prototype equipped with a UV-B barrier film (Figure 4d). The advantage of the irrigation system proposed in this work compared to the traditional systems is the simplicity of the manufacturing with the absence of assembled parts. Furthermore, it is possible to adapt the porosity of the permeable structure according to the water permeability of the soil and the needs of the plant to be irrigated [58,59,60].

#### 3.3.1. Morphology Characterization by Scanning Electron Microscope (SEM) and X-ray Tomography

**Figure 5:**
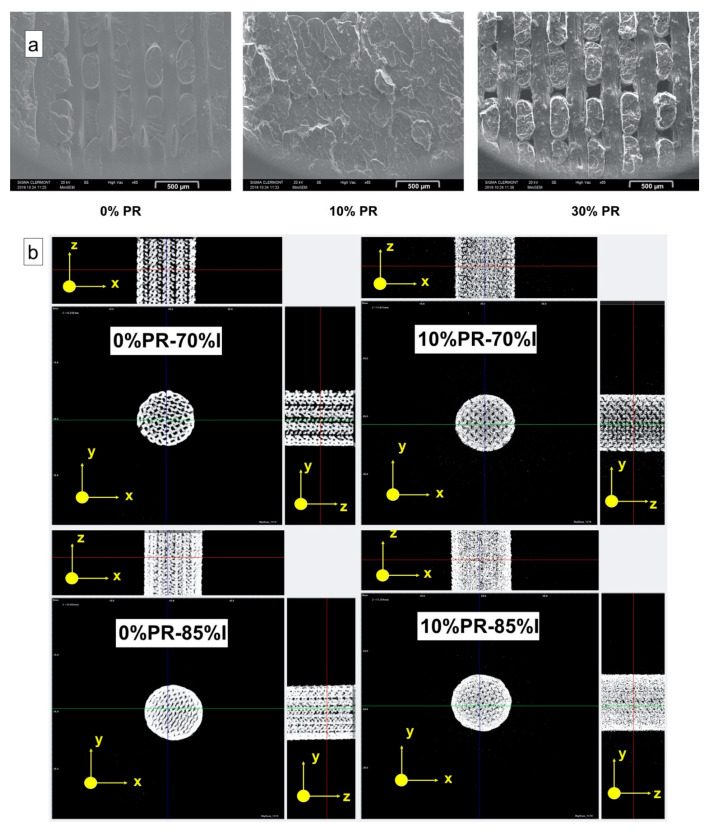
Scanning electron microscope (SEM) pictures of the fracture cross-section of the tensile specimens (**a**) and axonometric projections of the permeable part 3D printed at different ID percentages obtained with X-ray tomography (**b**).

The morphological analysis of the fractural-cross section of the printed samples subjected to the tensile test was carried out with a SEM. Figure 5a shows the results related to the samples with 0%, 10%, and 30% pozzolan content. It was observed that the amount of microvoids, created among the printed layers due to the lack of cohesion, changed with the amount of pozzolan content. In particular, the sample with 10% of pozzolan presented the lowest rate of microvoids, highlighting better cohesion between the layers in comparison with the samples at 0% and 30% of pozzolan content. The improved interlayer cohesion observed for the composite at 10% of pozzolan content can be due to the higher thermal conductivity thanks to the presence of the pozzolan. The thermal conductivity allows a higher overall thermal profile during cooling, improving the welding between the raster lines. However, in the case of the composites with 30% of pozzolan content, the macromolecular diffusion between the layers was slowed down by the presence of a high amount of filler, causing an important increase of viscosity and consequently less welding between the raster lines [46,61,62,63].

Moreover, an X-ray tomography analysis was performed, and the porosity values were evaluated for all samples. The results showed that the highest total porosity value was depicted for the samples at 30% of pozzolan content while the lowest one was presented for the composites at 10% of pozzolan (Table 6). Therefore, the X-ray results were in accordance with the observation made by the SEM images. The X-ray tomography was conducted to also observe and analyze the mesostructure of the permeable part printed at different infill densities of the self-watering pot. Figure 1b and Figure 5b show the 3D models of the longitudinal sections of the printed parts obtained from the tomographic reconstruction and the isomeric projection graphs, respectively. As can be seen, the largest fraction of voids could be found in the case of the sample printed at 70% ID (Figure 1b), which was reduced with the addition of the pozzolan. The total porosity values are shown in Table 6 where the higher values were observed for the part printed with the pure matrix, thus also confirming, in this case, that the pozzolan improved the interlayer cohesion. Furthermore, it is interesting to observe from the isomeric projection images (Figure 5b), a radial distribution of the porosity, which intensified at the center of the piece where the white portion had less density. This result can be traced back to the print path of the nozzle. In fact, on the perimeter of the object, the print path was subjected to sudden changes of direction, causing a sudden jerk of the nozzle. This phenomenon affects the deposition of the material, which is distributed more on the external region of the piece due to longer dwell time.

#### 3.3.2. Mechanical Properties

The mechanical properties such as Young’s modulus, ultimate strength, ultimate strain, and impact strength are shown in Table 7. These results are related to the samples obtained by injection molding and 3D printing (simultaneously and sequentially). The samples obtained by injection molding showed an increase in rigidity by increasing the pozzolan content in the polymer matrix, characterized by an increase in Young’s modulus and a reduction in the ultimate strength and ultimate strain. A decrease was observed in the Charpy impact with the addition of the pozzolan filler. It is well known that the addition of fillers in polymeric matrices leads to the reinforcement of the material with the formation of micro-defects and stress concentration, which reduces the breaking strength of the composite [64]. In the case of samples obtained with 3D printing, the addition of the pozzolan had a reinforcing effect by observing an increase in Young’s modulus compared to the samples printed with the pure polymer. Furthermore, in the case of 3D printed samples, there was an increase in the ultimate strength and impact strength that instead did not occur in the case of samples obtained by injection molding. However, there was a reduction in the properties for the composites charged at 30% of pozzolan, and the printing of the specimens for the impact tests failed in the case of simultaneous printing due to the clogging of the nozzle. No differences were found between the samples printed simultaneously and sequentially. This printing strategy allowed us to analyze the effect of the delay time on the cohesion between the layers. In previous studies, the effect of the deposition delay time of the layers during the printing was analyzed where the results showed that better cohesion appeared when the polymer had a slow crystallization rate [46,48]. In this study, using simultaneous or sequential printing mode (different deposition delay time) did not show significant differences in mechanical properties. This result could be connected to the similar crystallization kinetics observed for the used composites.

### 3.4. Study of the Functionality of the Smart Irrigation System

#### 3.4.1. Liquid Water Transport Properties

**Figure 6:**
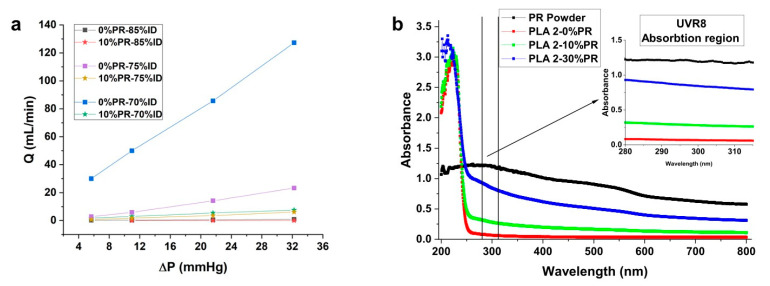
Volumetric water flow as a function of the pressure gradient applied along the permeable part of the self-watering pot (**a**), UV–Vis absorbance spectra of the printed film at different percentages of PPW (**b**).

The self-watering capacity of the 3D printed pot was assessed by measuring the volume flow as a function of the infill densities and the percentage of pozzolan. Figure 6a shows the graph of the volumetric flow rate as a function of the pressure gradient along the permeable part that was printed at different infill densities. Furthermore, the values for the PLA2 at 0 and 10% of pozzolan content are reported. As can be seen, the graph showed a linear dependence of the water flow on the pressure gradient for all the analyzed samples. Interestingly, the pozzolan allowed for the reduction of the water flow through the porous volume. In particular, at the highest pressure gradient value, for the samples printed at 70% of infill density, there was a reduction in the flow rate of about 120 mL/min between the samples printed with 0% of pozzolan and samples printed with 10% of pozzolan content. This behavior can be linked to the capacity of the 10% pozzolan composites to improve the intermolecular diffusion between the layers during printing, reducing the microvoids, as observed from the morphological analysis.

#### 3.4.2. UV-B/UVR8 Barrier Properties

The UV–Vis spectroscopy was used to measure the absorption spectrum of the 3D printed film that had been designed to protect the plants from UV-B radiation in the wavelength range equal to 280–315 nm, which has been identified as photo activators of plant UVR8 photoreceptors. Figure 6b shows the curves related to the absorption of the pozzolan powder used as a filler in this study, and for the films made by PLA2 with different percentages of pozzolan. Considering the region of wavelength between 280 and 315 nm, the absorption curve increased with increasing pozzolan content. This result represents an advantage for the UV-B barrier and activators of the UVR8 photoreceptors [65,66]. Below 295 nm, plants do not receive UV-C radiation from the sun due to the presence of ozone in the atmosphere, therefore the barrier produced by the film is helpful to growth. Concerning the UV-A wavelength range, the film had a certain barrier that attenuated with increasing wavelength. In conclusion, these results show the possibility of using 3D printing to elaborate thin films as part of complex geometry and having functional properties such as barrier properties.

## 4. Conclusions

A new eco-composite based on natural pozzolan waste was studied. Three different PLA grades named PLA1, PLA2, and PLA3 were used as a polymer matrix to produce the composites. The obtained sample was subjected to thermal and rheological characterization. The results showed that the composites produced with PLA2 (5% D-lactide content) had intermediate properties among the different studied composites. In particular, the thermal behavior did not show significant variations following the addition of the pozzolan, retaining its crystallization kinetics. Instead, for PLA1, the pozzolan did not significantly affect the thermal behavior of the pure matrix, while for PLA3, the pozzolan acted as a nucleating agent. Concerning the rheological behavior, the viscosity of the composites increased with the increase in the pozzolan content, and the normal force increased during the crystallization phenomenon. However, PLA2 showed intermediate behavior in the evolution of the normal force and viscosity. The highest increase in the normal force was observed for the PLA3/PPW composites; in contrast, the PLA1/PPW did not show a significant increase. Consequently, given its optimum properties, the composite based on PLA2 was chosen to produce a filament for material extrusion fabrication. Mechanical tensile and impact tests were conducted on the 3D printed and injected specimens. The results showed that the pozzolan acts as a reinforcement for the composites for both the printed and injected samples. However, in the case of 3D printed pieces, the 30% pozzolan composite involved less cohesion between the layers in comparison to the 10% pozzolan one, as depicted by X-ray tomography and SEM analysis. Consequently, the mechanical properties were highest for the composite with 10% of pozzolan. Finally, a smart irrigation pot with a porous structure for the control of the water flow and a UV-B barrier film was designed and printed. The effect of the pores on the passage of water was measured by evaluating the water flow rate as a function of the pressure gradient along the permeable part. The measurements showed that the presence of the pozzolan reduced the passage of water. The porosity of the permeable part was analyzed with X-ray tomography showing a radial distribution of pores on the cross-section of the considered part. Regarding the barrier film, the analysis of UV–Vis spectroscopy showed that the presence of the pozzolan can play a role as a UV-B absorber in the range of 280–315 nm, consequently, the results confirm the possibility of printing a complex piece with a thin film with specific functionalities.

## Figures and Tables

**Table 1:** General properties of the PLA used in this study.

Polymers	Elastic Modulus	Mw	T_m_	MFI	D-Enantiomers
	MPa	KDa	(°C)	10 g/min (190 °C/2.16 Kg)	%
PLA 1	630	130	150	9	7
PLA 2	2690	180	150	6	5
PLA 3	3200	200	170	4	1.5

**Table 2:** Extrusion conditions for the compound process.

Heating Zone	Z1	Z2	Z3	Z4	Z5	Z6	Z7	Z8	Z9
Temperature (°C)	70	162	163	162	163	165	166	165	170
Screw rate (rpm)	350								
Mass flow (Kg/h)	10								

**Table 3:** 3D printing process parameters.

Parameter	Value
Nozzle diameter (mm)	0.4
Nozzle temperature (°C)	215
Layer thickness (mm)	0.15
Bed temperature (°C)	60
Printing speed for the 1st layer (mm/s)	20
Printing speed for the other layers (mm/s)	80

**Table 4:** Injection molding process parameters.

Barrel Heating Zones (°C)	Z1	Z2	Z3	Z4	Z5
	85	175	170	170	170
Mold temperature (°C)	40				
Colling time (s)	20–25				
Molded volume (cm^3^)	50				
Injection flow (cm^3^/s)	30–35				
Holding pressure (bar)	60				
Holding time (s)	10				
Dosing screw speed (m/min)	15				
Injection pressure (bar)	60–70				

**Table 5:** Thermal parameters obtained with differential scanning calorimetry (DSC) analysis.

Polymer	T_g_	T_m_	T_cc_	x_c_	ΔH_c_	t_1/2_
Unities	°C	°C	°C	%	J/g	s
PLA 1 0%PR	60	151	127	1	-	2640
PLA 1 10%PR	59	151	-	1	-	2620
PLA 1 30%PR	60	151	127	3	-	2621
PLA 2 0%PR	60	151	109	14	1.9	244
PLA 2 10%PR	60	151	109	10	1.8	245
PLA 2 30%PR	60	151	109	11	1.4	240
PLA 3 0%PR	62	170	117	11	1.7	395
PLA 3 10%PR	63	170	98	38	9.4	173
PLA 3 30%PR	63	170	-	36	17.0	128

**Table 6:** Morphological parameters obtained with X-ray tomography.

Samples	Specific Surface of the Voids	Closed Porosity	Open Porosity	Total Porosity
	1/mm	%	%	%
Tensile samples				
0%PR	4.1	1.0	2.0	3.1
10%PR	2.1	0.4	0.1	0.5
30%PR	8.2	4.6	3.6	8.1
Self-watering samples				
0%PR–70%ID	13.9	1.8	33.5	34.7
10%PR–70%ID	7.5	2.4	6.3	8.5
0%PR–75%ID	6.9	1.0	10.6	11.6
10%PR–75%ID	6.2	2.6	4.1	6.6
0%PR–85%ID	7.6	3.6	5.1	8.7
10%PR–85%ID	3.7	2.2	0.3	2.5

**Table 7:** Mechanical characterization results: Young’s modulus, ultimate strength, ultimate strain, and impact strength.

Injected Samples	Young’s Modulus	Ultimate Strength	Ultimate Strain	Impact Strength
	(MPa)	(MPa)	(%)	(KJ/m^2^)
0%PR	2654 ± 77	56 ± 2.3	2.6 ± 0.1	18 ± 2.2
10%PR	3038 ± 140	51 ± 2.1	2.2 ± 0.1	17 ± 1.2
30%PR	3762 ± 250	51 ± 1.8	1.8 ± 0.1	14 ± 1.0
Samples printed Simultaneously				
0%PR	2400 ± 22	42 ± 2.1	2.3 ± 0.2	14 ± 0.6
10%PR	3140 ± 78	51 ± 1.6	2.1 ± 0.1	16 ± 1.2
30%PR	3100 ± 92	30 ± 2.7	1.3 ± 0.3	-
Samples printed Sequentially				
0%PR	2440 ± 16	46 ± 1.9	2.4 ± 0.1	14 ± 1.7
10%PR	3230 ± 60	51 ± 2.2	2.0 ± 0.2	15 ± 0.6
30%PR	3100 ± 90	34 ± 2.5	1.4 ± 0.3	8 ± 0.5

## Data Availability

The data presented in this study are available on request from the corresponding author.
